# The effect of the Progressive Goal Attainment Program on cognitions, perceptions, and work participation of workers with chronic health problems: study protocol for a randomized controlled trial

**DOI:** 10.1186/s13063-022-06698-8

**Published:** 2022-09-09

**Authors:** Mariska de Wit, Hendrika P. Zijlstra, Carel T. J. Hulshof, Sylvia J. van der Burg-Vermeulen, Angela G. E. M. de Boer

**Affiliations:** 1 Amsterdam UMC location University of Amsterdam, Public and Occupational Health, Coronel Institute of Occupational Health, Meibergdreef 9, Amsterdam, The Netherlands; 2 Amsterdam Public Health Research Institute, Societal Participation & Health, Amsterdam, The Netherlands; 3 Zorg van de Zaak, Utrecht, The Netherlands

**Keywords:** Progressive Goal Attainment Program, PGAP, Intervention, Work participation, Workability, Cognitions, Perceptions, Chronic health problems, Workers, Netherlands

## Abstract

**Background:**

Cognitions and perceptions of workers with chronic health problems, such as catastrophizing thoughts and fear-avoidance beliefs, can negatively influence work participation. The Progressive Goal Attainment Program (PGAP) is an intervention developed in Canada with the aim of decreasing limiting cognitions and perceptions and increasing work participation. The objective of this protocol article is to describe the design of a randomized controlled superiority trial to study whether PGAP is effective in decreasing limiting cognitions and perceptions and increasing workability and work participation of workers with chronic health problems in the Netherlands.

**Methods:**

This study is a randomized controlled superiority trial with two (parallel) groups, in which workers on sick leave are randomly assigned to an intervention group (PGAP intervention) or to a waiting-list control group (care as usual). The PGAP intervention consists of a maximum of 10 weekly individual sessions provided by a trained PGAP professional in which the worker learns about staying active, planning activities, and setting goals. Participants in this risk-targeted behavioral activation intervention also learn to be more aware of their cognitions and perceptions and learn about solution-focused problem-solving skills in challenging situations. The primary outcome is the degree of catastrophizing. Secondary outcomes are other personal cognitions and perceptions (e.g., expectations regarding return to work, self-efficacy), health symptoms (e.g., fatigue, depression), work participation (e.g., sick leave status, work hours), and other work-related outcomes (e.g., workability, quality of working life).

**Discussion:**

Although PGAP shows positive effects in Canada, we do not know whether this intervention is effective in the Netherlands. This study is the first randomized controlled trial to test the effect of PGAP on limiting cognitions and perceptions and on work participation of workers with chronic health problems in the Netherlands. If PGAP is effective it could be implemented in the Netherlands in order to stimulate workability and work participation of workers.

**Trial registration:**

The protocol of this study is registered in the Netherlands Trial Register (NL9832) in October 2021.

**Supplementary Information:**

The online version contains supplementary material available at 10.1186/s13063-022-06698-8.

## Background

Because of better treatments, an increase in life expectancy, and the increased retirement age, the number of people of working age with chronic health problems is rising [1–3]. Having chronic health problems can have a negative impact on workability and return to work (RTW) after sick leave [4–6]. Important factors that can influence work participation of workers with chronic health problems are cognitions and perceptions of workers, such as catastrophizing thoughts and fear-avoidance beliefs [7]. Results of previous studies show that catastrophizing thoughts can lead to delayed recovery, an increase in the duration of work absence, work disability, and functional limitations [7–10].

In a scoping review, interventions were identified that were aimed at changing cognitions and perceptions and increasing work participation [11]. The Progressive Goal Attainment Program (PGAP) is a promising intervention with the aim to decrease limiting cognitions and perceptions and to increase work participation [12, 13]. This risk-targeted behavioral activation intervention, which consists of a maximum of 10 sessions, focuses on important cognitions and perceptions, such as fear-avoidance beliefs, catastrophizing thoughts, RTW expectations, or self-efficacy. PGAP is aimed at different groups of patients, i.e. patients with pain, depression, posttraumatic stress disorder, cancer, and other chronic health problems. The intervention can be provided by different occupational health (OH) professionals, such as occupational physicians (OPs) or OH practitioners, after completing a short training program [13].

Studies show positive effects of PGAP on decreasing catastrophizing thoughts, decreasing fear-avoidance beliefs, and increasing RTW in Canada [14–16]. The intervention is so far implemented in other countries such as the USA [17], Australia [18], Ireland [19], and South Africa [20] in which positive effects of PGAP were demonstrated. In the USA, participation in PGAP increased the activity of participants, reduced fear, and improved the ability to pursue goals [17]. In South Africa, participation in PGAP increased self-efficacy and decreased pain catastrophizing and fear-avoidance beliefs [20]. In Australia, participation in PGAP resulted in a reduction of depressive symptoms and fatigue symptoms [18].

Because the empirically supported effects of PGAP are positive, it is aimed at a broad group of patients and it can be provided by different OH professionals, PGAP is a promising intervention to change cognitions and perceptions and improve work participation. However, it is unknown whether PGAP is effective in the Dutch context in changing cognitions and perceptions and stimulating work participation. Because occupational healthcare differs across countries, it may be possible that the effect of PGAP in comparison to care as usual in the Netherlands differs from other countries that studied the effects of PGAP [21]. Besides, although different studies demonstrate positive effects of PGAP, the effects of PGAP are not studied among workers with different chronic health problems in a randomized controlled trial yet. Therefore the objective of this protocol article is to describe the design of a randomized controlled superiority trial with two parallel groups to study whether PGAP (1) is effective in decreasing limiting cognitions and perceptions and (2) is effective in increasing workability and work participation of workers with chronic health problems in the Netherlands. We hypothesize that participants who participate in PGAP score lower on limiting cognitions and perceptions, such as catastrophizing, fear-avoidance, perceived injustice, and perceived disability than participants who do not participate in PGAP. We also hypothesize that participants who participate in PGAP score higher in work ability, have a higher chance to RTW and have more working hours than participants who do not participate in PGAP.

## Methods

The Medical Ethics Review Committee of the Amsterdam UMC – location Academic Medical Center, University of Amsterdam, approved the randomized controlled trial described in this protocol (2021_231). All procedures performed in the trial involving human participants will be in accordance with the ethical standards of the institutional and/or national research committee and with the 1964 Helsinki declaration and its later amendments or comparable ethical standards. The trial is registered in the Netherlands Trial Register (NL9832). This study protocol is written in accordance to the Standard Protocol Items: Recommendations for Interventional Trials (SPIRIT) guidelines (Additional file 1) [22].

### Study setting

This project was initiated by the Department of Public and Occupational Health, Coronel Institute of Occupational Health of the Amsterdam UMC location University of Amsterdam, which is located in the Netherlands. The Netherlands has about 17,4 million inhabitants. The participants will be recruited by OPs and OH practitioners from a national occupational health service (OHS), which has multiple branches spread over the Netherlands. The core business of the OHS is to offer services to different organizations with the goal to increase the health of workers, promote sustainable employability, prevent sick leave, and offer support for RTW after sick leave. The task of the OP in the Netherlands is to prevent work-related diseases and to support workability and RTW after sick leave. The OH practitioner collaborates with the OP and works in task delegation of the OP to reduce the work pressure of the OP. Both OPs and OH practitioners have consultations with workers to promote workplace health, to prevent sick leave, or to support RTW. Six OH practitioners from the OHS will also be the PGAP providers (“coaches”) in this study. The six OH practitioners were selected by the director of the OHS (HZ) by convenience sampling. The OH practitioners were eligible to become a PGAP coach if they could understand and speak Dutch and English.

### Study design

**Fig. 1:**
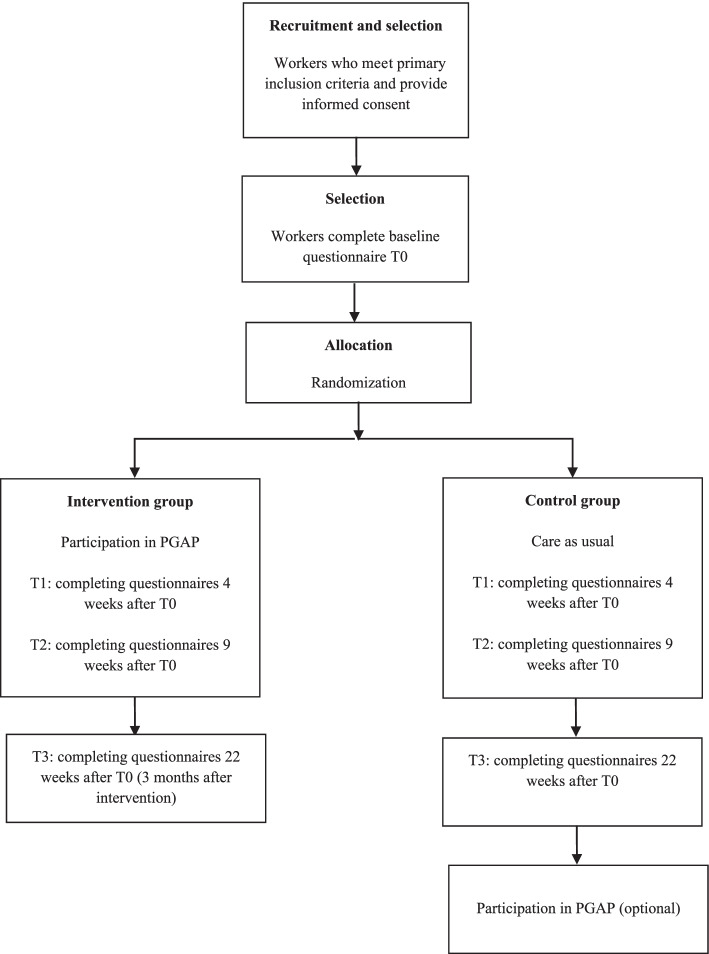
PGAP study flow-chart

This study is a randomized controlled superiority trial with two parallel groups, in which participants are randomly assigned to an intervention group (PGAP intervention) or a waiting-list control group (care as usual) with an allocation ratio of 1:1. An OP (CH), insurance physician (SV), and the director of an OHS (HZ) were involved in the development in this protocol and study design. They have extensive experience with workers with health problems and know a lot about the practice of OPs and OH practitioners. Besides, the developers of PGAP, who have a lot of experience with PGAP and studying the effects of PGAP were consulted for advice. The design and different measurements of this study are presented in Fig. 1. The schedule of enrollment, interventions, and assessments are presented in Table 1.

**Table 1:**
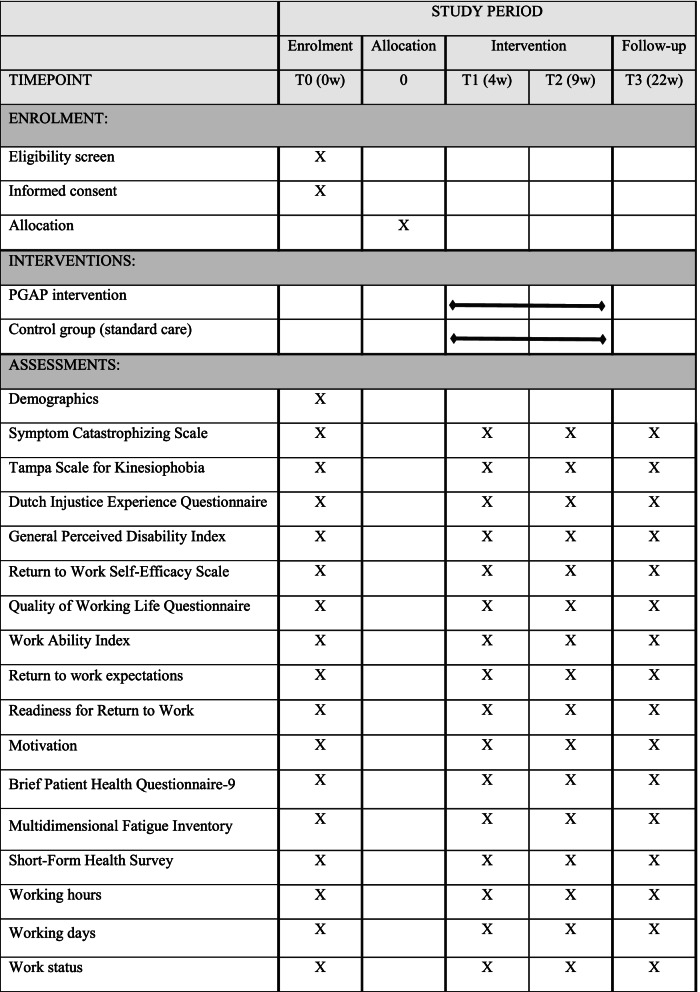
Schedule of enrollment, interventions, and assessments

### Participants

Persons are eligible for inclusion if they are workers between 18 and 65 years old. To participate, persons have to be on sick leave for at least 3 weeks or have frequent spells of sick leave, defined as 3 or more times a year. Eligible participants need to be able to speak, read and write in Dutch and need to be in paid employment. They are eligible if they experience functional limitations associated with their mental or physical health problems. In addition, they need to have at least one cognition or perception that can limit work participation; catastrophizing thoughts, perceived injustice, fear-avoidance beliefs, or disability beliefs. Whether the workers have these limiting cognitions and perceptions will be indicated by a score above the threshold on different questionnaires which are described in the outcome measures section of this protocol.

### Sample size

Results of power analysis (*α* = .80, *p* < .05) show that at least 25 participants per group are needed to detect a change in post score of 2.4 points on the primary outcome catastrophizing as measured with the Symptom Catastrophizing Scale (SCS; SD = 3.0). The standard deviation of 3.0 points and the change of 2.4 points on the SCS are based on a previous study on the effects of PGAP by Moore et al. [23]. On the basis of previous research on the effect of PGAP, we expect that we will lose 15% of the participants in follow-up [24]. Therefore we aim to randomize at least 60 participants; 30 in the control group and 30 in the intervention group.

### Recruitment procedure

Participants will be recruited by OPs and OH practitioners from a large national OHS in the Netherlands. The OP or OH practitioner assesses whether the worker is possibly eligible for participation. The OP or OH practitioner assesses (1) whether the worker is on long-term or frequent sick leave, (2) whether he or she experiences functional limitations associated with his or her mental or physical complaints, and (3) whether the worker possibly has cognitions or perceptions that may limit workability or work participation. When the OP or OH practitioner thinks PGAP is a suitable intervention for the worker, he or she asks if the worker is interested in participating in the PGAP project. The OP or OH practitioner gives the participant a leaflet with information about the project. If the worker is interested and gives permission, the OP or OH practitioner will send the contact details of the worker to the researchers. Next, the researchers send the worker the information letter about the PGAP project and an informed consent form. Workers will also be able to ask questions about the PGAP project during an online meeting with one of the researchers and will watch an introduction video about PGAP. If the worker is not interested in participation, the OP or OH practitioner asks whether the worker wants to provide the reason for this, but the worker is not obliged to provide a reason. When the worker is interested, has signed the informed consent form, and has sent it to the researcher, he or she receives the first screening questionnaire which can be completed online. With the screening questionnaire, we will assess whether the worker really has limiting cognitions and perceptions. Only then, the worker meets the inclusion criteria and can participate in PGAP. The screening questionnaire will also be used as the T0 measurement.

In order to motivate workers to participate in this study and to remain participants throughout the study, all participants will have the opportunity to participate in PGAP without costs and all participants will receive a gift card of 20 euros after finishing the study.

### Allocation

After completing the screening questionnaire, participants will be randomized in the intervention group or in the waiting-list control group in which participants receive care as usual, with an allocation ratio of 1:1. Randomization will be performed by one author (MdW) using Castor (https://www.castoredc.com). The OPs, PGAP coaches, and participants will not be blind to the condition of the participants. After randomization, all participants are labeled with a research code consisting a unique number in order to stay anonymous.

### Intervention

PGAP consists of a maximum of 10 weekly one-to-one sessions of a trained PGAP coach with a worker [13]. PGAP can be provided face-to-face, online, or by telephone [13, 25]. For this study, all sessions will be provided online through Zoom. The sessions have a duration of one hour. During the sessions, the PGAP coach uses different techniques to reduce limiting cognitions and perceptions in order to stimulate (work) participation. Participants are encouraged to talk about the difficulties they experience as a result of their health complaints and learn to be more aware of their thoughts in challenging situations and how to react in challenging situations. In addition, they learn about the importance of staying active, planning activities, and setting goals and are encouraged to resume discontinued activities. An introduction video is used to inform the worker about the procedures and objectives of the PGAP intervention. All workers will be given a workbook with information about the importance of sleep, social activities, and exercise, in which also activities can be scheduled. The workbook is also used to guide participants through exercises aimed at for example reducing catastrophizing thoughts and perceived injustice. The workbook will be translated in Dutch and the introduction videos will be subtitled in Dutch. A professional translation service, a worker with chronic health problems, the developers of PGAP, and the authors of this article will all be involved in the translation of the PGAP materials from English into Dutch. The content of the 10 sessions of PGAP is summarized in Table 2.

**Table 2:** Content PGAP sessions [13]

Session	Content
Session 1	– Use of disclosure to establish a therapeutic relationship – Discussing activities that were performed by the client before the start of his/her complaints – Instructions on how to use the client workbook
Session 2	– Introduction to activity planning – Re-establishing pre-injury activity structure – Introduction to walking routine
Session 3	– Setting short and long-term activity goals – Planning activity involvement in relation to goals
Session 4	– Techniques targeting disability beliefs – Mid-treatment evaluation to see whether return to work is possible
Session 5	– Feedback about the mid-treatment evaluation – Introduction to thought monitoring to target catastrophic thinking
Session 6	– Exposure techniques to facilitate re-engagement in previously avoided activities
Session 7	– Continued application of techniques introduced in sessions 5 and 6
Session 8	– Applying task decomposition techniques to feared activities of the workplace
Session 9	– Problem-solving challenges to resumption of occupational activities – Final evaluation
Session 10	– Evaluation feedback and discharge planning

From “A Psychosocial Risk-Targeted Intervention to Reduce Work Disability: Development, Evolution, and Implementation Challenges” by M.J.L. Sullivan, H. Adams, and T. Ellis, 2013, *Psychological Injury and Law, 6,* 253

The main goal of PGAP is to decrease limiting cognitions and perceptions and to increase work participation. PGAP finishes if the worker has returned to work. Therefore, it is possible that workers do not need all 10 sessions. After the fourth session, the worker completes an evaluation questionnaire to see whether limiting cognitions and perceptions are decreased. The PGAP coach and worker discuss, with the input of the scores on the evaluation questionnaire, whether RTW is possible. If limiting cognitions and perceptions are decreased and RTW is possible, the fifth session of PGAP is the last session for the participant. If RTW is not possible, the participants will continue the PGAP sessions until RTW is possible. The PGAP intervention will be provided by OH practitioners after completing a training to become a PGAP coach. The training consists of watching 9 webinars, participating in a 2-day training workshop to acquire the skills necessary to deliver PGAP, and passing a final exam.

During the trial, meetings will be scheduled between the PGAP coaches and an experienced PGAP supervisor in which PGAP coaches can discuss the progress of their cases or any problems they might experience during the trial. To obtain information about the compliance of the PGAP coaches to the PGAP protocol, all PGAP coaches will complete a short checklist after each session. In this checklist, the PGAP coaches can indicate if important goals per session are met and which topics during the sessions were discussed.

The participants in the control group will receive care as usual, which could differ between the participants depending on their health issues and duration of sick leave. After the participants in the control group complete the final assessment, they will have the opportunity to participate in PGAP. The participants in both the intervention and control groups are allowed to participate in other interventions or care during the trial. Whether they participate in other types of care will be assessed in the questionnaires.

### Outcome measures

#### Primary outcomes

The primary outcome is catastrophizing thoughts as measured with the Dutch SCS [23]. The Dutch SCS is a modified version of the Dutch Pain Catastrophizing Scale [26] in which items are modified to make them better suited for individuals who do and who do not experience pain, but other mental or physical health problems. The questionnaire has 7 items with a three-point scale from “never” to “often.” The final score on the SCS can range from 0 to 14, in which a higher score indicates having more catastrophizing thoughts. Previous studies showed that the SCS is a reliable (Cronbach’s alpha .81–.89) and valid questionnaire to measure catastrophizing thoughts [23].

#### Secondary outcomes

Secondary outcomes are other cognitions and perceptions than catastrophizing, health symptoms, and different work-related outcomes.

Fear-avoidance beliefs will be measured with 5 items from the Dutch version of the Tampa Scale for Kinesiophobia (TSK) [27, 28]. The five items can be answered on a three-point scale from “do not agree” to “completely agree.” The final score can range from 0 to 10 in which a higher score indicates more fear avoidance. The Cronbach’s alpha of the Dutch TSK was .77 in previous studies [27].

Perceived injustice will be measured using a modified version on the Dutch Injustice Experience Questionnaire (IEQ) [29, 30]. The modified IEQ contains 5 items that can be answered on a three-point scale from “never” to “often.” The final score can range from 0 to 10 in which a higher score indicates more perceived injustice. Previous studies showed that the IEQ is valid and reliable (Cronbach’s alpha .92) for measuring perceived injustice [29, 30].

Perceived Disability will be measured using the Dutch version of the General Perceived Disability Index (GPDI), which is a modified version of the reliable (Cronbach’s alpha .86) and valid Pain Disability Index [31–33]. In the GPDI the wording of the items is modified to refer to symptoms of the conditions, instead of referring only to pain. The questionnaire contains 5 items that can be answered on an eleven-point scale from “no disability” to “total disability.” The final score can range from 0 to 50 in which a higher score indicates more perceived disability.

Self-efficacy in RTW will be measured using the Dutch version of the RTW Self-Efficacy (RTW-SE) Scale [34]. This questionnaire has 11 items with a six-point scale from “totally disagree” to “totally agree.” The RTW-SE score is computed by taking the mean score over the 11 items, in which a higher score indicates more self-efficacy in RTW. Previous studies indicated that the RTW-SE is a reliable (Cronbach’s alpha .90–.96) and valid measure for self-efficacy in RTW [34].

Quality of working life will be measured using the Dutch version of the valid and reliable (Cronbach’s alpha .91) Quality of Working Life Questionnaire (QWLQ) [35]. This questionnaire has 23 items with a six-point scale from “totally disagree” to “totally agree.” The overall QWQL score is calculated with a standardized score ranging from 0 to 100. A higher score indicates better quality of working life.

Workability will be measured using the Dutch version of the Work Ability Score and two additional questions of the reliable and valid Work Ability Index (WAI) [36–38]. The Work Ability Score is the worker’s self-assessment of his/her current ability ranging van 0 to 10. In the other two questions of the Work Ability Index, the workers rate his/her workability in relation to the physical and mental work demands. The questions can be answered on a five-point scale from “very poor” to “very good.”

RTW expectations will be measured using the questions: “What are your chances of being fully able to work one month from now?” and “What are your chances of being fully able to work three months from now?” The questions can be answered on a scale from 0 to 100%.

Readiness for RTW will be measured with the Dutch Readiness for RTW scale (RRTW) [39, 40]. This questionnaire has 21 items with a five-point scale from “strongly disagree” to “strongly agree.” Twelve of these items are only completed by participants who did not RTW and 9 items are only completed by participants who returned to work. The scores of this questionnaire indicate in which stage the worker is when it comes to returning to work: pre-contemplation, contemplation, preparation for action-self-evaluative, preparation for action-behavior, uncertain maintenance, and proactive maintenance. The Cronbach’s alpha for these dimensions varied from .65 to .82 in previous studies [39].

Motivation will be asked with one statement that can be answered on a five-point scale from “totally disagree” to “totally agree.” The statement for participants who returned to work is “I am motivated to stay at work.” The statement for participants who did not RTW is “I am motivated to RTW”.

Depressive symptoms will be measured with the Dutch version of the Brief Patient Health Questionnaire-9 (PHQ-9) [41]. The questionnaire contains 9 items which can be answered on a four-point scale from “not at all” to “nearly every day”. In addition, there is a tenth item in which the participant can indicate how difficult the problems of the participant have been lately. The final score can range from 0 to 27 in which a higher score indicates more severe depressive symptoms. The Cronbach’s alpha for the PHQ-9 varied from .86 to .89 in previous studies and was proven to be a valid questionnaire for measuring depressive symptoms [41].

Fatigue will be measured with the Dutch version of the Multidimensional Fatigue Inventory (MFI) [42]. The questionnaire contains 20 items which can be answered on a five-point scale from “yes, that is true” to “no, that is not true.” The final score can range from 20 to 100 in which a higher score corresponds with higher levels of fatigue. The MFI is a valid and reliable questionnaire for measuring fatigue (Cronbach’s alpha .84) [42].

Health-related quality of life will be measured using the Dutch version of the valid and reliable (test-retest correlation .76–.89) 12-item Short-Form Health Survey (SF-12) [43, 44]. The questionnaire contains 12 items related to physical and mental health. The final score can range from 0 to 100 in which a higher score corresponds with a better physical and mental health functioning.

Work participation will be measured by asking the participants about their current contract hours, about the mean days and hours they currently work during a week, and by asking them whether they have modified work. In addition, we ask the participants about their sick leave status.

To check the readability and face validity of the questionnaires, a worker with chronic health problems completed all the questionnaires and was asked for feedback.

### Data collection and management

Data will be collected with electronic questionnaires which can be completed via the website of LimeSurvey (www.limesurvey.org). LimeSurvey is approved by the Amsterdam UMC to collect data and LimeSurvey never discloses data publicly, nor transfers any data to any third party without consent. In LimeSurvey data is stored in a separate database with a separate username and password for each LimeSurvey Cloud instance. The data will be collected at four different time points: during screening (T0), 4 weeks after screening (T1), 9 weeks after screening (T2), and 22 weeks after screening (T3). Data of the participants will be stored in a secured map on the local network of the Amsterdam UMC. The secured map is only accessible to the researchers of the Amsterdam UMC and to the members of the Clinical Monitoring Center of the Amsterdam UMC who will monitor this study. If data is transferred electronically from the researchers to the PGAP coaches, it will be sent through a secured file sender. Only project group members will have access to all the data collected. PGAP coaches have only access to the scores on the questionnaires of the participants they coach.

### Statistical analyses

Statistical analyses will be conducted in SPSS statistics 26. All analyses will be described in a statistical analysis plan, which can be provided upon request. All analyses will be performed according to the intention-to-treat principle. Descriptive statistics will be used to describe the control and intervention groups.

Generalized Linear Mixed Models will be used to study the effects of PGAP on the primary outcome catastrophizing as measured with the SCS at Times T1, T2, and T3, using baseline catastrophizing as a covariate.

Generalized Linear Mixed Models will be used to study the effects of PGAP on the following secondary outcomes, which are all continuous variables at Times T1, T2, and T3: score RTW self-efficacy scale, score QWLQ, score on WAI, score RTW Expectations, score RRTW scale, score on motivation scale, score TSK, score IEQ, score GPDI, score PHQ-9, score MFI, score SF-12, the mean number of working hours, and the mean number of working days. In the analyses, we will adjust for the baseline scores on these measures.

Mixed effects logistic regression will be used to study the effects of PGAP (intervention or control) on the secondary outcome work status (binary variable: back to work or on sick leave) at T1, T2, and T3.

**Fig. 2:**
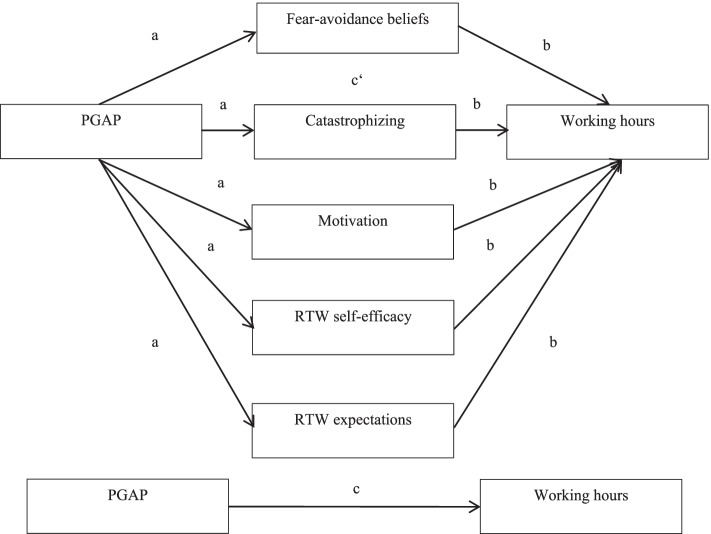
Model mediators following the approach of Baron and Kenny [45]

To examine whether decreases in catastrophizing or fear-avoidance beliefs or increases in RTW self-efficacy, motivation, or RTW expectations serve as mediators for the effects of PGAP on changes in working hours we will use the approach of Baron and Kenny (Fig. 2) [45]. We will analyze whether PGAP influences working hours (c), potential mediators (a), and whether the potential mediators influence working hours (b). Finally, we will analyze whether the magnitude of the effect of PGAP on working hours reduces in magnitude when we include the mediators in the model (c’).

Because the participant completes all questionnaires online and it is not possible to skip items, we do not expect missing items. However, it is possible that participants drop out and do not complete all questionnaires because of the longitudinal design of this study. We took this into account in our power analysis. Besides, the intention-to-treat analysis will be conducted to evaluate whether there are differences between the participants that completed all questionnaires and the participants who dropped out.

### Data monitoring

The Clinical Monitoring Center of the Amsterdam UMC will be responsible for monitoring. The monitors are in no other way involved in this study and there is no conflict of interest. Monitoring visits will take place during the study and are carried out on the basis of a monitoring plan. After each monitor visit, the monitor writes a report in which the findings are mentioned. Further details about its charter can be provided upon request.

Because no risks are associated with participation in this study, no interim analyses are conducted and no stopping guidelines are formulated. Although no (serious) adverse events are expected, adverse events will be reported to the sponsor, which will report them to the Medical Ethics Review Committee. No auditing is planned. Amendments to the research protocol will be reported to the Medical Ethics Review Committee for approval.

### Ethics and dissemination

All participants will receive verbal and written information about the study and will sign an informed consent before participation. Data will only be available for project group members and the assigned PGAP coaches. We aim to publish the results of the trial in an open-access journal, so that results will become available for healthcare professionals and other interested groups. However, participants’ names and contact details will not be used in articles.

### Ancillary and post-trial care

Although no risks are associated with participation in PGAP, ancillary and post-trial care will be provided for participants who suffer sustained harm due to their involvement in this trial. All participants will be able to participate in PGAP.

## Discussion

The purpose of the randomized controlled trial described in this protocol article is to study whether PGAP is effective in changing cognitions and perceptions and increasing workability and work participation of workers with a chronic health problems in the Netherlands.

Previous studies have emphasized the importance of cognitions and perceptions for work participation [7–10]. Also, OH professionals recognize the importance of cognitions and perceptions, which makes these factors important targets for intervention [46]. Although different foreign studies have emphasized the positive effects of PGAP, this intervention is not available in the Netherlands yet. Because occupational healthcare differs across countries, it is important to study if PGAP is also effective in the Netherlands in comparison to the care as usual in the Netherlands [21]. This study is the first attempt to translate PGAP into the Dutch context and to study the effects of this intervention. If PGAP is effective in changing limiting cognitions and perceptions and improving work participation in the Netherlands, PGAP can be recommended by Dutch OH professionals.

### Strengths and limitations

A strength of this study is that we study the effects of PGAP in a randomized controlled trial, which is a robust study design for examining the effects of interventions. During this study, we work closely together with the Canadian developers of PGAP (MS, HA) who also have experience with studying the effects of this intervention in Canada. Besides, we collaborate with a large national OHS in the Netherlands, which is necessary to recruit participants from different companies across the country.

The study has also some possible limitations. First, it is not possible to blind the researchers, PGAP coaches, and participants to the condition of the participants and outcome data, which might influence the results of this study. Second, in this study, we will not distinguish between different chronic health problems, although it might be possible that the effects of PGAP differ across different health problems. All participants will be recruited from one OHS which may limit the generalizability of the research findings. Finally, the follow-up period of this study is relatively short. Therefore long-term effects of PGAP will not be evaluated.

## Conclusion

This study is the first randomized controlled trial to test the effect of PGAP on cognitions and perceptions and on work participation of workers with a chronic health problems in the Netherlands. If PGAP is effective it could possibly be implemented in the Netherlands to stimulate work participation of workers with a chronic health problems. We expect the results of this study to be available in 2023.

### Trial status

Recruitment will start in September 2022 and is expected to continue until February 2023.

Protocol version 4.0, April 2022.

## Supplementary Information

**Additional file 1.** SPIRIT checklist.

## Data Availability

Data will be available from the author on reasonable request.

## Abbreviations

FMI: Multidimensional Fatigue Inventory
GPDI: General Perceived Disability Index
IEQ: Injustice Experience Questionnaire
OPs: Occupational physicians
OH: Occupational health
OHS: Occupational health service
PGAP: The Progressive Goal Attainment Program
PHQ-9: Brief Patient Health Questionnaire-9
QWLQ: Quality of Working Life Questionnaire
RRTW: Readiness for RTW scale
RTW: Return to work
SCS: Symptom Catastrophizing Scale
SF-12: 12-item Short-Form Health Survey
SPIRIT: Standard Protocol Items: Recommendations for Interventional Trials
TSK: Tampa Scale for Kinesiophobia
WAI: Work Ability Index
